# Resident Physicians are at Increased Risk for Dangerous Driving after Extended-duration Work Shifts: A Systematic Review

**DOI:** 10.7759/cureus.4843

**Published:** 2019-06-05

**Authors:** Nicole T Mak, Jennifer Li, Sam M Wiseman

**Affiliations:** 1 Surgery, University of British Columbia, Vancouver, CAN; 2 Surgery, St. Paul’s Hospital & University of British Columbia, Vancouver, CAN

**Keywords:** residency, traffic accidents, occupational health, sleep deprivation, inattentive driving, medical education

## Abstract

Background: Resident physicians often work longer than 24 consecutive hours with little or no sleep. A systematic review of the literature was conducted to investigate the risk of resident physician motor vehicle collisions (MVC), and dangerous driving, after extended-duration work shifts (EDWS).

Material and methods: A keyword search was performed for original research articles evaluating any aspect of driving safety following EDWS for the resident physician population. Two authors independently reviewed articles for inclusion. Subsequent independent data abstraction and quality appraisal were carried out. Five articles met the study inclusion criteria.

Results: The quality of the evidence was low to very low. Results were not pooled due to study heterogeneity. Residents reported between 2.3 to 3.8 hours of sleep during EDWS. Three survey-based studies identified an increased risk of MVCs and falling asleep at the wheel after EDWS. One study associated weekly cumulative sleep hours lost with the risk of falling asleep while driving. Both driving simulation and survey studies linked EDWS with MVCs. Notably, a driving simulation study found an increase in crash frequency in male residents post-EDWS. Additionally, a survey reported that the risk of an MVC post-EDWS increased by 16.2% per shift worked in a month.

Conclusion: The period following EDWS is associated with an increased risk of potentially life-threatening driving safety risks for resident physicians. These observations warrant careful consideration. They suggest that there is a need for greater awareness and action in order to avoid the occupational and public health risks of driving after EDWS.

## Introduction

Extended-duration work shifts (EDWS) enable hospitals to ensure adequate physician coverage of medical services outside of regular daytime working hours. In teaching hospitals, this has traditionally involved resident physicians remaining in hospital overnight until the next day, and working greater than 24 consecutive hours with little sleep. Several factors have led to the adoption of extended duration shifts in hospital medicine: a sense of responsibility for the patient, the unique learning opportunities presented by overnight call, and the availability of personnel and funding [1]. Concerns for occupational health and public safety associated with work-related fatigue have been raised by the medical education community and the public [2-5]. Specific concerns include but are not limited to: driving safety, needle-stick injuries, increased substance abuse, and impairment of knowledge acquisition [6].

Concerns regarding patient safety were the impetus for a change in work hour restrictions in the United States [1,5-8]. In 2000, Williamson and Feyer reported on the effect of work-related sleep deprivation and compared it to blood alcohol concentrations (BAC). They found that over 17 sequential hours of wakefulness resulted in a change in performance similar to that of being at the legal limit for BAC [9]. Subsequently, Arnedt et al. found that repetitive episodes of acute sleep deprivation (one night without sleep or with inadequate sleep) by medical trainees result in neurobehavioural effects equivalent to a BAC roughly between 0.04 and 0.05 g% [10].

While the majority of Canadian teaching hospitals still rely upon a 30 hour work hour restriction and 24-hour call shifts for trainees, a limit of 16 consecutive work hours has been imposed in the United States [5,11]. As a result of these work hour restrictions, a shift towards night-float systems has occurred in the United States and in some Canadian provinces. Day shifts are scheduled for 8 to 10 hours, while night shifts now range between 14 to 16 hours in duration. Even though shiftwork has decreased the number of hours of acute sleep deprivation, there are ongoing concerns for patient safety, physician health and the cumulative impact of chronic fatigue [7,10].

Work hour restriction is a sensitive and controversial topic within the medical education system [1,5,11-14]. One very important aspect to consider when this issue is reviewed is resident driving safety. Evidence has suggested that there is a tendency towards increased dangerous driving and motor vehicle collisions (MVC) in sleep-deprived residents [6,15-20]. The objective of this review is to systematically evaluate the current literature in order to determine whether resident physicians demonstrate an increased risk for dangerous driving after extended-duration work shifts.

## Materials and methods

An electronic literature search was conducted using the following databases: Embase (1974 to February 2018), PubMed (1946 to February 2018), Cochrane Database (2005 to February 2018), and Ovid Medline (1946 to February 2018). The search strategy was formulated according to the Population/Intervention/Comparison/Outcome (PICO) approach (Table 1).

**Table 1:** Population, intervention, comparison, and outcome (PICO) framework

Population	Resident Physicians (Any Specialty)
Intervention (Exposure)	Extended-duration Work Shifts (> 16 hours)
Comparison	Day Shifts of Normal Duration (8 hours)
Outcome	Traffic Accidents or Dangerous Driving Behaviours

The following keyword search for titles and abstracts in Ovid Medline was designed to identify relevant articles: (“Traffic” OR “Road accident*” OR “Car*” OR “Automobile*” OR “Drive” OR “Driving”) AND (“Resident*” OR “Intern*”) AND (“Work hour*” OR “Workload” OR “On call” OR “Shift work” OR “Sleep deprivation”). The search strategy was adapted for and repeated in each reference database. In the Embase and Medline databases, the subject headings “automobile driving”, “internship and residency”, and “workload” was also applied to the keyword search.

Firstly, duplicates were manually removed by a single reviewer. A total of 638 abstracts were screened by two independent reviewers for subsequent full-text review. Abstracts were included only if they reported original research, either observational or experimental. Study inclusion criteria included: observational or experimental design published as a full-text article; population including resident physicians (also known as “residents”, “interns” or “housestaff”); evaluates any outcome relating to automobile driving after extended-duration work shifts. The study exclusion criteria were: articles not containing original research (e.g. news or opinion articles, review articles); duration of work shift less than 16 hours, or not clearly defined; publications in a language other than English or French. A single reviewer completed a handsearch of the complete reference list of all 13 included full-text articles, as well as relevant review articles.

Two independent reviewers used the Newcastle-Ottawa Scale (NOS) to assess risk of bias in three categories: selection, comparability, and outcome [21]. The GRADE criteria was then applied to rate the overall quality of each outcome. Data extraction and quality assessment were performed using common data extraction tables and the aforementioned standardized criteria.

## Results

Search results and characteristics of included studies

**Figure 1:**
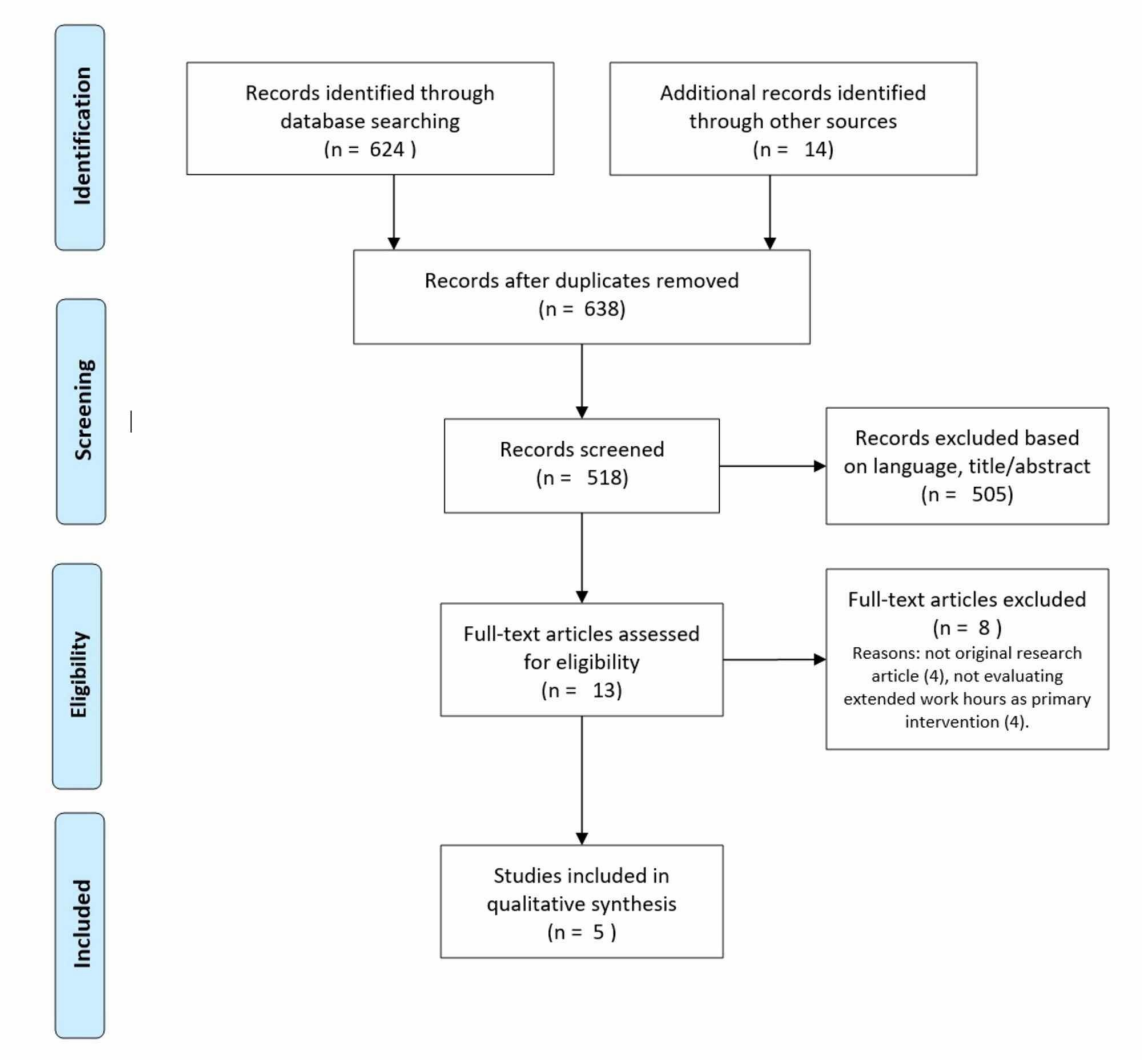
PRISMA diagram for article screening and selection process performed by two independent reviewers PRISMA: Preferred reporting items for systematic reviews and meta-analyses.

The literature search yielded a total of 1019 abstracts. After removal of duplicates, and title and abstract review, thirteen full-text articles were evaluated. Eight full-text articles were excluded. Five articles that reported the results of five unique studies met study inclusion criteria. Figure 1 depicts the screening and selection process that was carried out by two independent reviewers. Selected studies are listed and their characteristics are summarized in Table 2. Three observational studies using survey-type data were identified [18-19,22]. Two of these were retrospective survey studies, while one was a prospective repeated-measures study that took place over a one-year period. The remaining two studies employed driving simulators and a within-subjects design to compare resident driving performance in the absence and presence of post-EDWS sleep deprivation [16-17].

**Table 2:** Characteristics of included studies EDWS: extended-duration work shift.

Author (Year)	Study Location	Study Type	Population	EDWS Definition
Barger et al. (2005) [18]	USA	Prospective repeated-measures survey	2737 First Year Residents	- Duration: >24 hr - Frequency: 3.9 +/- 3.7 shifts per month. - Average hours of sleep on duty: 2.6 +/- 1.7 hr
Marcus et al. (1996) [19]	USA	Retrospective survey	61 Pediatrics Residents and 74 attending physicians	- Duration: >24 hr - Frequency: 1:4 shift:day - Average hours of sleep on duty: 2.7 +/- 0.9 hr
O’Grady et al. (2012) [22]	Australia and New Zealand	Retrospective survey	659 Surgery Residents	- Duration: >24 hr - Frequency: 1:4.4 shift:day - Average hours of sleep on duty: unknown
Ware et al. (2006) [17]	USA	Experimental study, within-subjects design.	22 Medicine Residents and 1 Student	- Duration: 19 to 24 hr - Frequency: 1:3 to 1:4 shift:day - Average hours of sleep on duty: Men: 3.3 +/- 1.2 hrs Women: 3.8 +/- 0.9 hrs
Tornero et al. (2012) [16]	Spain	Experimental study, within-subjects design.	25 Emergency Medicine Residents	-Duration: >24 hr - Frequency: unknown - Average hours of sleep on duty: 2.34 +/- 0.64 hr

Study quality assessment

Details on the quality analysis conducted using the NOS are summarized in Table 3.

**Table 3:** Quality assessment for included studies by the Newcastle-Ottawa Scale (NOS)

Author (Year)	Selection (maximum 4 stars)	Comparability (maximum 2 stars)	Outcome (maximum 3 stars)	Total (maximum 9 stars)
Barger et al. (2005) [18]	***	*	*	*****
Marcus et al. (1996) [19]	**	*		***
O’Grady et al. (2012) [22]	**	*		***
Ware et al. (2006) [17]	*	*	***	*****
Tornero et al. (2012) [16]	*	*	***	*****

All studies were subject to participation bias. Five out of nine was the maximum number of stars assigned to each study using the NOS. Concerns regarding bias were raised with respect to study population sample selection and outcome assessment. The two non-observational studies were limited by small population sample sizes. Four out of five studies utilized within-person comparisons. All studies are limited by reporting bias for exposure or outcome. Determination of exposure and non-exposure was carried out entirely by participant self-reporting. Only Ware et al. and Barger et al. used actigraphy or direct activity observation, respectively, to quantify actual sleep hours on-duty. All survey-based studies relied on participants’ recall of events for a time period that ranged from one month to three years, potentially biasing the temporal relation between EDWS and driving incidents. The overall quality of evidence was evaluated according to GRADE criteria. Evidence for motor-vehicle collision risk and risk of falling asleep at the wheel, was low and very low quality, respectively. Evidence was considered low quality due to a lack of a robust study design and the risk of recall and reporting bias.

Study setting and participants

Though all studies evaluated aspects of driving safety, participants’ practice setting varied. Three out of five studies were conducted at American post-graduate training sites [17-19]. One study was conducted among surgical trainees in Australia and New Zealand, and another studied emergency medicine residents in Spain [16,22]. The specialty of participants varied between studies, and residents in medical and surgical training programs were both represented. Among the survey-type studies, Marcus et al. focussed on pediatric interns, O’Grady et al. included only surgical trainees, and Barger et al. include trainees in both medical and surgical specialties. The two experimental driving simulation studies evaluated medical residents and emergency medicine residents, respectively [16-17]. Four of the studies were each comprised of a cohort of medical trainees exclusively. The vast majority of these trainees were residents, only one study included a single medical student. O’Grady et al. included attending physicians as a control group. The attending physicians were presumed not to be exposed to the same degree of sleep deprivation as trainees during EDWS [19].

Summary and comparison of study findings

Extended-duration Work Shifts and Reported Fatigue/Sleepiness

A complete description of participants’ typical EDWS schedule was only provided by 3 studies [17-19]. The reported frequency of EDWS, duration of EDWS, and number of hours of sleep on call varied between studies (Table 2). The average number of hours of sleep obtained during an overnight call shift ranged between 2 and 4 hours. The manifestation of sleep deprivation as “fatigue” or “sleepiness” post-EDWS was measured by 2 studies. Ware et al. employed standard measures (Visual Analogue Scale) to quantify sleepiness post-EDWS, while O’Grady et al. created a Likert-type questionnaire to evaluate participants’ degree of fatigue [17,22]. Trainees reported significantly greater sleepiness and fatigue after a night of being on call. Interestingly, after completing a driving simulation task post-EDWS, trainees reported becoming even more sleepy on the Visual Analogue Scale. Further demonstrating the association between work-related sleep deprivation and fatigue is the observation that residents who reported being “almost always fatigued” have 7.1 +/-3.3 hrs of cumulative sleep deficit when compared to 3.5 +/-3.0 hrs of cumulative sleep deficit reported by residents who “seldom feel fatigued” [22].

Risk of Falling Asleep at the Wheel While Driving

The most common outcomes evaluated by the included studies were the risks of falling asleep at the wheel while driving, and the risk of MVCs (Table 4).

**Table 4:** Findings of included studies EDWS: extended-duration work shift; MVC: motor-vehicle collision; NR: not reported.

Author (Year)	Falling Asleep at the Wheel	Risk of MVC
Barger et al. (2005) [18]	- OR falling asleep at the wheel if working > 5 EDWS vs. 0 EDWS = 2.39 (95% CI, 2.31 to 2.46)	- OR crash after EDWS = 2.3 (95% CI, 1.6 to 3.3) - OR near-miss after EDWS = 5.9 (95% CI, 5.4 to 6.3) - Increase in risk for crash on commute home per EDWS = 16.2% (95% CI 7.8 to 24.7%)
Marcus et al. (1996) [19]	- 44% of residents fell asleep at the wheel at traffic lights vs. 12.5% of attending physicians fell asleep at the wheel at traffic lights (p<0.001)	- 40% of reported MVCs occur when residents are post-call
O’Grady et al. (2012) [22]	- Residents who never fall asleep at the wheel have less total sleep hours lost - Momentary dozing while driving is more likely if >5.5 h of weekly sleep loss hours (p<0.05)	NR
Ware et al. (2006) [17]	NR	- Significant increase in crash frequency after overnight call in male residents only.
Tornero et al. (2012) [16]	NR	- No significant difference in overall test results after EDWS compared to test results after 7 hours of rest.

The risk of falling asleep at the wheel was measured using self-reporting questionnaires, requiring residents to recall up to 3 years of their driving history. One study compared the driving history of attending physicians and residents [19]. This study found that attending physicians were significantly less likely to report falling asleep while driving. Attending physicians were assumed to have significantly less fragmented sleep during their call shifts, and thus to have a lesser sleep deficit. A clearer exposure-response relationship between sleep deprivation and falling asleep while driving was suggested by the findings of the two other survey-based studies [18,22]. Barger et al. found that residents who worked a greater number of EDWS per month had a greater risk of falling asleep while driving. In their study of Australasian surgical trainees, O’Grady et al. found a correlation between falling asleep behind the wheel and the total number of sleep hours lost.

Risk of Motor Vehicle Collisions

The risk of being in an MVC was evaluated by both experimental and observational study designs (Table 4). In a study that compared residents and attending physicians, residents were found to have a greater total number of reported motor vehicle collisions, and more moving traffic citations [19]. In their prospective study, Barger et al. established an exposure-response relationship between EDWS and MVCs. They found that the odds of MVCs and near-miss events are increased post-EDWS. Similarly, each EDWS worked per month increased the residents’ risk of an MVC during the post-EDWS drive by 16.2%. Only one of two studies that used an experimental approach was able to relate crash frequency to the post-EDWS state. Both experimental studies measured driving performance using standardized driving simulation tests. These tests recorded variables such as reaction time, driving speed, vehicle speed variance, and lane position variance. The driving simulator employed by Ware et al. quantified the crash frequency, and found a significant increase in crashes only in male residents [17]. Though Tornero et al. did not measure crash frequency, their experiments did not measure a difference in driving performance parameters when comparing the post-EDWS state to the rested state [16].

## Discussion

Five studies that evaluated residents’ driving risk following EDWS were identified and included in our systematic review. Although included studies were conducted on three different continents, in all cases, the work structures for trainees included work shifts that lasted longer than 24 hours. Two main themes emerged from our review: that residents are at risk for falling asleep behind the wheel while driving after long work shifts, and that work-related fatigue may contribute to increased MVCs and near-miss driving events. Only one small simulation-based study did not identify a difference in resident driving behaviour after EDWS. It is inferred here that the reason for unsafe driving behaviour is the irregular nature of resident physicians’ working hours. Overall, despite considerable heterogeneity in study approach, the studies that were evaluated in this review suggest that there is a direct relationship that exists between motor vehicle collisions, and sleep hours lost by trainee physicians.

The belief that sleep deprivation is largely to blame for the risks to residents’ occupational health and patient safety has led to reactionary measures by regulating bodies such as the ACGME (American Council for Graduate Medical Education), and provincial residency unions throughout Canada [5,11-12,14,23]. Duty hour restrictions have been established, limiting the number of consecutive hours worked or the total weekly hours worked. Duty hour restrictions require a fundamental change in the work schedule with a move towards shift work. Shift work itself has also been found to have a concerning impact on motor-vehicle operation [24]. A survey-based study conducted by Steele et al. on more than one-thousand emergency medicine trainees revealed that 80% of MVCs occur after night work shifts, and that the number of collisions was directly proportional to the number of night shifts worked in a month [20]. More recently, Huffmyer et al. conducted high-fidelity driving simulation experiments on anesthesiology residents working 6 consecutive “night-float” shifts within the context of a schedule that followed the ACGME work hour restrictions [15]. These restrictions placed a cap on the number of consecutive hours worked, and the total number of hours worked per week. Their findings of decreased reaction times, and increased attention lapses after a week of working night shifts suggest that the current restructured work schedules have continued to negatively affect the safety of post-call motor vehicle operation.

Providing overnight patient care is unavoidable in medicine, and is also an essential and important part of resident training. A variety of different frameworks have been proposed to ensure patients’ continuity of care and to meet residents’ educational needs. Day float, night float, and shift work structures are all employed to meet these important demands. Driving safety is only one of the many considerations that are required to inform policy changes with regards to managing resident work structures. It is currently unclear how different scheduling structures impact resident neurocognition and clinical judgment. Studies generally have not demonstrated that the current ACGME duty hour restriction regulations lead to improvements in either patient care or medical education. However, these restrictions have been reported to have a positive impact on residents’ reported overall well-being [13]. As this is a complex area that must address many competing interests, it is difficult to build sufficient evidence to strongly support the adoption of one particular scheduling structure over another [6,11,23]. Determining whether the primary cause of impaired neurocognition and increased risk of personal injury is acute sleep deprivation, chronic fatigue, circadian rhythm disruption, or some combination of these, could inform the safe restructuring of resident and physician on call structures. Further complicating matters is the reality that skill sets and training demands of specialties vary considerably. Identifying an ideal framework that is universally applicable to all physicians seems unlikely. Moreover, it has been shown in both the long-distance trucking and health care industries that there is significant variation in individual susceptibility to fatigue from both EDWS and shift work [20,25-27]. Perception studies that have been performed in Canadian medical and surgical residents more recently have reported conflicting observations. Specifically, medical residents responded positively to duty hour restrictions, while surgical residents felt duty hour regulations negatively impacted their surgical education and patient care [4].

Safe operation of a motor vehicle following EDWS is a common concern for residents within all medical specialties. The American Academy of Sleep Medicine (AASM) recognizes drowsy driving as a serious public health concern [27]. Industries with long work hours or shift work, such as healthcare, are encouraged to prevent excessive employee sleepiness, and provide education on fatigue management strategies and the risks of drowsy driving. Individual employees’ tolerance of sleep debt may be accounted for by increasing awareness of the symptoms of sleepiness. The AASM recommends prevention strategies for individuals at risk that include: pulling to the side of the road when drowsy, seeking alternate drivers, and ensuring adequate sleep prior to driving long distances. In the aviation industry, whereas in healthcare, error-free performance is critical, fatigue risk management systems based upon sleep science have been developed. For long-haul flights, strategic in-flight sleep is utilized by pilots as a risk mitigation strategy [28]. Additionally, fatigue risk management systems are also considered an important matter of shared responsibility between flight team members. By contrast, in the case of resident physicians, the ethical responsibility of safe motor vehicle operation after EDWS is left up to the individual. Awareness and avoidance are reasonable initial preventative strategies. At the institutional level, in order to optimize occupational health and well-being during residency and post-residency, education focused on responsible driving post-EDWS should be considered a very important priority.

An important limitation with regards to the interpretation of data for this review was the quality and heterogeneity of the included studies. Any temporal associations between EDWS and uncommon outcomes such as MVCs and traffic citations should be interpreted within the context of the high risk of recall and reporting biases given the predominance of self-reported survey-type studies. Four countries are represented in the included studies with three countries being primarily English-speaking. It is possible that the results reported were influenced by the local regulations and cultural perceptions of the communities in which they were conducted. Similarly, the findings here may not apply to the training practices of all medical communities. Nevertheless, the overall directionality of study outcomes was consistent and concerns about the implications of EDWS and sleep deprivation are shared in many countries [3-4,29].

## Conclusions

In summary, driving during the period following EDWS that lasts longer than 16 consecutive hours poses significant safety risks for resident physicians. This was observed in both the records of residents’ actual driving history, and from simulated driving challenges. A link between long work hours, sleep disruption, fatigue and motor vehicle collisions is suggested. The deleterious effects of sleep disruption extend beyond the hospital walls; physicians’ personal safety and the safety of the general public are at risk. These findings identify an important occupational health issue. They highlight the need for further research to better understand the relationship between work hours and occupational health. This will further inform future awareness campaigns and policy-making which should aim to mitigate health and safety risks such as driving after EDWS.
